# Supplementary material for: A Critical View of the Application of the APEX Software (Aqueous Photochemistry of Environmentally-Occurring Xenobiotics) to Predict Photoreaction Kinetics in Surface Freshwaters
Source: Molecules. 2019 Dec 18;25(1):9. doi: 10.3390/molecules25010009 (PMC7017383; doi:10.3390/molecules25010009)
Supplement: Supplementary file 1 [file molecules-25-00009-s001.zip › 3.2.4_gcc-4.4.0.zip!/3.2.4_gcc-4.4.0/3.2.4_gcc-4.4.0/doc/octave/html/interpreter/The-_003ccode_003econtinue_003c_002fcode_003e-Statement.html]

The @code{continue} Statement - Untitled


Next: The <code>unwind\_protect</code> Statement,
Previous: The <code>break</code> Statement,
Up: Statements


---

### 10.7 The `continue` Statement

The `continue` statement, like `break`, is used only inside
`for` or `while` loops. It skips over the rest of the loop
body, causing the next cycle around the loop to begin immediately.
Contrast this with `break`, which jumps out of the loop altogether.
Here is an example:

```
     # print elements of a vector of random
     # integers that are even.
     
     # first, create a row vector of 10 random
     # integers with values between 0 and 100:
     
     vec = round (rand (1, 10) * 100);
     
     # print what we're interested in:
     
     for x = vec
       if (rem (x, 2) != 0)
         continue;
       endif
       printf ("%d\n", x);
     endfor
```

If one of the elements of vec is an odd number, this example skips
the print statement for that element, and continues back to the first
statement in the loop.

This is not a practical example of the `continue` statement, but it
should give you a clear understanding of how it works. Normally, one
would probably write the loop like this:

```
     for x = vec
       if (rem (x, 2) == 0)
         printf ("%d\n", x);
       endif
     endfor
```
